# Vision-Based 6D Pose Analytics Solution for High-Precision Industrial Robot Pick-and-Place Applications

**DOI:** 10.3390/s25154824

**Published:** 2025-08-06

**Authors:** Balamurugan Balasubramanian, Kamil Cetin

**Affiliations:** 1 Department of Electrical and Electronics Engineering, Izmir Katip Celebi University, Cigli, 35620 Izmir, Türkiye; 2 Eren Brake Linings, Kemalpasa, 35170 Izmir, Türkiye; 3 Smart Factory Systems Application and Research Center, Izmir Katip Celebi University, Cigli, 35620 Izmir, Türkiye

**Keywords:** industrial robot arm, industrial camera, pick-and-place application, 6D pose estimation, analytics solution, LabVIEW, YOLO

## Abstract

High-precision 6D pose estimation for pick-and-place operations remains a critical problem for industrial robot arms in manufacturing. This study introduces an analytics-based solution for 6D pose estimation designed for a real-world industrial application: it enables the Staubli TX2-60L (manufactured by Stäubli International AG, Horgen, Switzerland) robot arm to pick up metal plates from various locations and place them into a precisely defined slot on a brake pad production line. The system uses a fixed eye-to-hand Intel RealSense D435 RGB-D camera (manufactured by Intel Corporation, Santa Clara, California, USA) to capture color and depth data. A robust software infrastructure developed in LabVIEW (ver.2019) integrated with the NI Vision (ver.2019) library processes the images through a series of steps, including particle filtering, equalization, and pattern matching, to determine the X-Y positions and Z-axis rotation of the object. The Z-position of the object is calculated from the camera’s intensity data, while the remaining X-Y rotation angles are determined using the angle-of-inclination analytics method. It is experimentally verified that the proposed analytical solution outperforms the hybrid-based method (YOLO-v8 combined with PnP/RANSAC algorithms). Experimental results across four distinct picking scenarios demonstrate the proposed solution’s superior accuracy, with position errors under 2 mm, orientation errors below 1°, and a perfect success rate in pick-and-place tasks.

## 1. Introduction

Industrial robot arms have been used for many years to increase the efficiency of modern manufacturing processes due to their high precision, repeatability, and speed capacities [1]. They are widely used in critical processes such as pick-and-place applications, assembly, packaging, and material handling, and these application capabilities directly affect the performance of production lines [2,3]. However, high-precision and high-accuracy positioning and manipulation of objects in dynamic and complex industrial environments pose significant challenges, especially in cases that require 6D (six-dimensional) pose estimation (three-dimensional position and three-dimensional orientation) [4,5]. In this study, the problem of picking metal plates located in different positions in a brake pad production line from the center of gravity with a vacuum–magnetic gripper integrated at the end point of the robot arm and placing them in certain empty slots with high precision is addressed. This process becomes complicated due to the different orientations and positions of the plates and requires high accuracy in both picking and placing operations. For example, if the gripper at the end point of the robot arm moves to the correct position but does not approach the metal plate with the correct orientation, it may not be able to grasp the metal plate from its targeted center point due to its weight, magnetic field effect, and, if any, its inclination relative to the production line. Therefore, a metal plate that cannot be picked with the desired position and orientation may not be correctly placed in the empty target slots. This problem emerges as a critical obstacle that needs to be solved in order to increase precision and minimize the margin of error in industrial production.

For estimating the 6D pose of objects, cameras are the most widely used technology in industrial applications [6], even if sensors such as lasers [7], IMU-based string encoders [8], and tactile sensors [9] are rarely used. In this study, a fixed eye-to-hand RGB-D camera is used because RGB-D cameras provide more accurate pose estimation by providing both color and depth information of objects simultaneously. In order to process the real-time images obtained from the camera and to compare the calculated 6D pose values, the actual dimensional parameters of the metal plates in the 3D computer-aided design (CAD) models are needed as reference values. To computationally estimate the 6D pose of objects, deep learning-based or traditional analytics-based solutions are often integrated with image processing techniques.

When we examine pioneering and innovative studies that have become reference points in the literature [10,11,12,13,14] on the estimation of objects’ 6D pose, the approaches to solving this problem are divided into two main categories: learning-based and non-learning-based techniques. Of the traditional non-learning-based 6D pose estimation techniques from the early years, the template matching-based and feature-based methods are still used today. LineMOD [15], the most well-known of the template matching methods, estimates the 6D pose of an object by comparing previously created 2D/3D templates from different viewpoints with the input image and selecting the best matching pose. Feature-based methods, other important methods that are non-learning-based, estimate the 6D pose by matching the 2D key points in the image with the corresponding 3D points in the object model and then solving the pose using geometric techniques such as perspective-n-points (PnP) and random sample consensus (RANSAC) [16]. For target objects exhibiting distinct textural features, traditional techniques such as the Hough Transform (HT) [17], Scale-Invariant Feature Transform (SIFT) [18], Speeded-Up Robust Features (SURF) [19], and Oriented FAST and Rotated BRIEF (ORB) [20] are often used for feature extraction and reliable pose estimation. In recent years, the estimation of objects’ 6D pose using these traditional techniques has been further developed and used in robotic grasping applications. In [21], the authors presented an approach for detecting and achieving high-accuracy 3D localization of multiple textureless rigid objects from RGB-D data. The authors of [22] presented a unique framework called Latent-Class Hough Forests for 3D pose estimation in situations that are very congested and obscured. Ref. [23] proposed a coarse-to-fine approach using only shape and contour data, selecting similar projection images to create many-to-one 2D–3D correspondences while emphasizing outlier rejection and leveraging geometric matching to guide pose estimation robustly. The authors in [5,24] developed the bunch-of-lines descriptor (BOLD) method to identify and match contour lines by fully using the geometric information of textureless metal objects in industrial applications. The study in [25] introduced a 3D point cloud pose estimation method using geometric information prediction to enhance accuracy and speed in robotic grasping of industrial parts by analyzing appearance characteristics and point cloud geometry. The study in [26] contributed a probabilistic smoothing method for stable object pose tracking in robot control using real and synthetic datasets. The study in [27] proposed a grasping pose estimation framework using point cloud fusion and filtering from a single-view RGB-D image.

Learning-based 6D pose estimation techniques include direct regression (e.g., PoseNet and SSD-6D), key point-based (e.g., PVNet and HybridPose), dense correspondence (e.g., DenseFusion and CosyPose), template matching (e.g., PPF-FoldNet), pose refinement (e.g., DeepIM and RePose), and differentiable rendering (e.g., DPOD and NeMo) methods. These methods rely on deep learning to predict object poses from RGB or RGB-D data with varying trade-offs in accuracy, robustness, and computational efficiency. Direct regression methods predict the 6D pose of the object from an input image in an end-to-end manner using deep neural networks, as demonstrated by PoseNet [28], which regresses camera pose from RGB images, SSD-6D [29], which combines detection and pose regression, and Deep-6DPose [30], which directly outputs key points and pose through a CNN. Key point-based methods predict the 6D pose by first detecting 2D key points (e.g., object corners or semantic points) in the image and then solving the pose using PnP algorithms, as seen in PVNet [31], which predicts vector fields for key point localization, and HybridPose [32], which combines key points with edge vectors and symmetry constraints for robust estimation. Dense correspondence methods establish per-pixel 2D–3D mappings between the input image and object model before computing the 6D pose, as demonstrated by DenseFusion [33], which fuses RGB and depth features for pixel-wise pose prediction, and CosyPose [34], which leverages dense matching for robust multi-object pose estimation in cluttered scenes. Template matching with deep features aligns input images with 3D object representations using learned feature descriptors, as exemplified by PPF-FoldNet [35], which encodes geometric Point Pair Features via deep learning, and Oberweger et al.’s method in [36], which employs CNNs to predict template matches for robust pose estimation under occlusion and textureless conditions. Pose refinement methods iteratively optimize an initial coarse pose estimate through learned correction mechanisms, as demonstrated by DeepIM [37], which uses iterative feature matching between observed and rendered images, and RePose [38], which employs differentiable rendering to analytically refine poses in a neural framework. Differentiable rendering-based methods optimize the estimation of 6D pose by comparing neural renderings of predicted poses without real images using gradient-based refinement, as exemplified by DPOD [39], which combines detection with differentiable silhouette matching, and NeMo [40], which aligns predicted and rendered surface normal maps for pose optimization.

Common features of hybrid methods are that they perform CNN-based feature extraction (key point, correspondence, and segmentation) in the learning phase and final pose optimization with PnP, RANSAC, ICP, or differentiable rendering in the geometric phase. Learning-based and non-learning-based hybrid approaches combine both the flexibility of deep learning and the robustness of traditional geometric methods in 6D pose estimation. Among the aforementioned methods, the most common hybrid methods and prominent examples are DenseFusion [33], PVNet [31], HybridPose [32], CosyPose [34], and DPOD [39]. Tekin et al. introduced YOLO6D in [41], a CNN that estimates 2D projections of 3D bounding box corners (You Only Look Once (YOLO) was first developed by [42]), enabling pose estimation via PnP via 2D–3D relations. Sundermeyer et al. introduced augmented autoencoders (AAEs) in [43], an extension of denoising autoencoders, which reduce the synthetic-to-real domain gap by training the model to be invariant to such discrepancies. Hodan et al. proposed a hybrid EPOS method in [44] that learns compressed 3D surface features and combines them with PnP and RANSAC. The authors in [45] proposed a region-based key point detection transformer, which uses set prediction and voting mechanisms to estimate the 6D pose in robotic grasping applications. Recent work by [46] demonstrated a vision-based pick-and-place system using an eye-in-hand camera and deep learning (YOLOv7 combined with GANs) to achieve real-time object recognition and precise robotic control in both simulated and real-world environments. In general, most of the above-mentioned geometry-based, learning-based, and hybrid methods focus on pose estimation itself without real-world applications. When we examine the studies mentioned above and the literature in general, we see that there are very few academic studies dealing with real problems in industrial applications. In our study, we address the problem of 6D pose estimation using a camera for a high-precision pick-and-place application of a metal brake pad plate with a robot arm in a real industrial application.

The learning-based and hybrid approaches mentioned in the literature usually require large datasets and intensive training processes, which can create difficulties in terms of efficiency and real-time performance in practical industrial applications. In this study, an analytical solution-based 6D pose estimation method with less computational complexity is presented, specially developed for a specific industrial task. Most of the studies mentioned above focus only on pose estimation itself and ignore real-world applications; therefore, academic studies dealing with real industrial problems are quite few. Existing studies generally do not take into account critical practical issues such as how the robot arm’s end-effector can fail operations such as grasping heavy metal plates from the center, even if it approaches the object in the correct position. Therefore, in this paper, the estimation of the 6D pose of an object encountered in a real industrial application is combined with robotic grasp planning to demonstrate the accuracy of the proposed method. In this respect, this study stands out as one of the rare studies that address vision-based high-precision 6D pose estimation and an industrial robotic pick-and-place application in an integrated manner.

In this study, we address the challenge of high-precision 6D pose estimation for a real-world industrial application by developing and validating a complete vision-guided robotic system. The main contributions of this study are summarized as follows:

- A lightweight, analytics-based 6D pose estimation method is developed and implemented in a real-world robotic pick-and-place system without requiring deep learning or large training datasets.
- The proposed method is experimentally validated on a Staubli TX2-60L robot arm integrated with an RGB-D camera and a vacuum–magnetic gripper in an actual brake pad production line.
- The system demonstrates high accuracy in real-time 6D pose estimation in four different object placement scenarios, achieving positional errors less than 2 mm and angular errors less than 1°.
- A comparative analysis is presented against state-of-the-art hybrid YOLO-based methods (e.g., YOLO + PnP/RANSAC), highlighting the accuracy, time-efficiency, consistency, and robustness of the proposed approach.

Although this study focuses on planar metallic brake pad plates due to their relevance in the specific industrial problem addressed, the proposed analytical 6D pose estimation approach is designed to be adaptable to broader object categories. Furthermore, while the current industrial setup benefits from relatively stable lighting and minimal occlusion, future adaptations may require addressing dynamic environmental factors such as varying illumination, partial object visibility, or complex geometries. The subsequent sections are organized as follows: Section 2 provides a detailed description of the robotic setup, focusing on the six-degree-of-freedom Staubli TX2-60L robot arm and the forward kinematic model created using the Denavit–Hartenberg (DH) method. Section 3 outlines the industrial camera setup, including the specifications of the Intel RealSense D435 camera, the calibration process for the camera and robot frames, and the LabVIEW software developed for image acquisition. Section 4 explains our proposed analytics-based solution, which uses the NI Vision library to process images and an inclination angle method to calculate the object’s 6D pose. For comparative analysis, Section 5 details the implementation of alternative hybrid methods, which use the deep learning-based algorithm YOLO-v8 for key point detection and various geometric algorithms (PnP/RANSAC) for pose estimation. Then, Section 6 presents the experimental studies, describing the pick-and-place application software and the four different test scenarios and discussing the results, which validates the high precision of our proposed analytical method against the hybrid approach. Finally, this study is concluded in Section 7.

## 2. Robotic Setup

**Figure 1:**
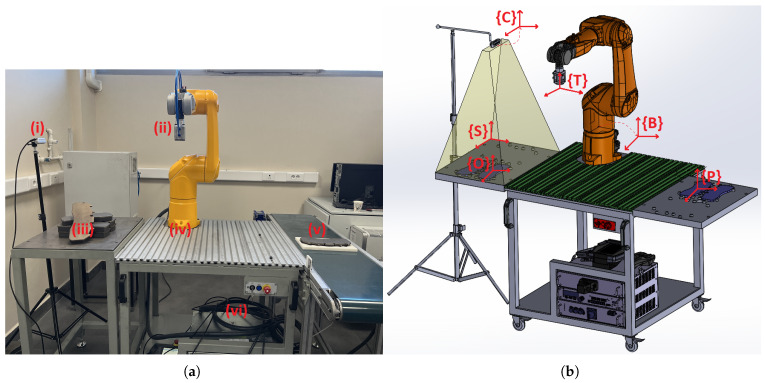
The overall setup structure consists of the following: (**a**) real system overview with (i) the RGB-D camera, (ii) the end-effector with the vacuum–magnetic gripper, (iii) the metal plates to be picked, (iv) the robot arm, (v) the empty slot in which to place the metal plates, and (vi) the CS9 controller unit. (**b**) Design and frame representations of overall system: {C} the camera frame, {S} the surface frame where metal plates are picked, {O} the object frame of the metal plate, {T} the tool frame of the end-effector of the arm, {B} the base frame of the arm, and {P} the empty slot frame where metal plates are placed.

In this study, a fixed eye-to-hand camera and a robot arm are used to place metal plates with random unknown positions in bulk in empty slots with specific poses in a brake pad production phase. For this real industrial pick-and-place application, a Staubli TX2-60L model robot arm and an Intel RealSense D435 camera are used. The general structure of the system setup is shown in Figure 1.

**Figure 2:**
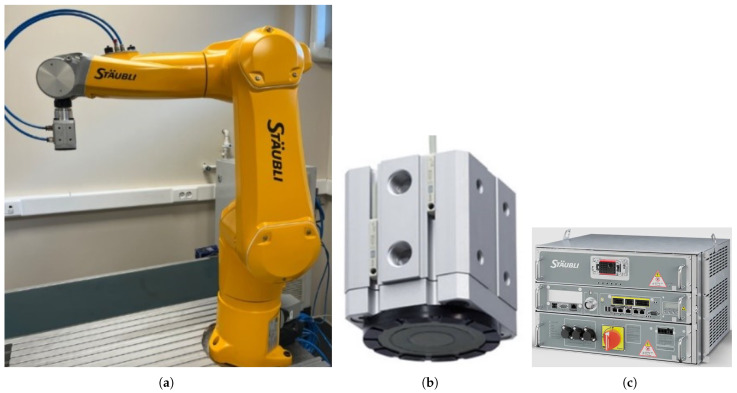
Robotic setup consisting of the following: (**a**) The Staubli TX2-60L model 6-DOF robot arm. (**b**) The vacuum–magnetic gripper. (**c**) The CS9 controller.

The Staubli TX2-60L model is a six-degree-of-freedom (DOF) robot arm, as shown in Figure 2a. The robot arm operates in a spherical workspace, has a reach of approximately 1 m, and can carry payloads of up to 3.7 kg. This TX2-60L model is equipped with 19-bit absolute encoders and is ready to operate without initialization. It has a repeatability of ±0.02 mm and is used for high-precision tasks such as assembly or parts handling. Due to its compact size, fast movement, and high repeatability, this robot is suitable for performing a wide range of operations and especially pick-and-place tasks that require speed and accuracy. In order to perform our pick-and-drop operations on the metal plates, a vacuum–magnetic gripper is mounted on the robot’s end-effector, as shown in Figure 2b, which can hold up to approximately 3.5 kg. The CS9 controller, as shown in Figure 2c, with an open architecture connects the robot to the computer via the Modbus TCP/IP industrial communication protocol and is used consistently in production because it has rapid integration.

In order for the robot to perform the task of picking up the metal plates, first the 6D pose of the objects is calculated with the RGB-D data coming from the camera. Then, the pose of the target object in Cartesian space is converted to the target end-effector pose of the robot arm using camera calibration calculations. In order for the robot to pick up the objects quickly and precisely with a correct trajectory strategy, the target trajectory positions of the robot’s end-effector are sent to the CS9 controller. The CS9 controller performs the movement of the metal plate held at the end-effector of the robot from the picked point to the place point using the encoder data in the joint motors and forward kinematic calculations.

**Figure 3:**
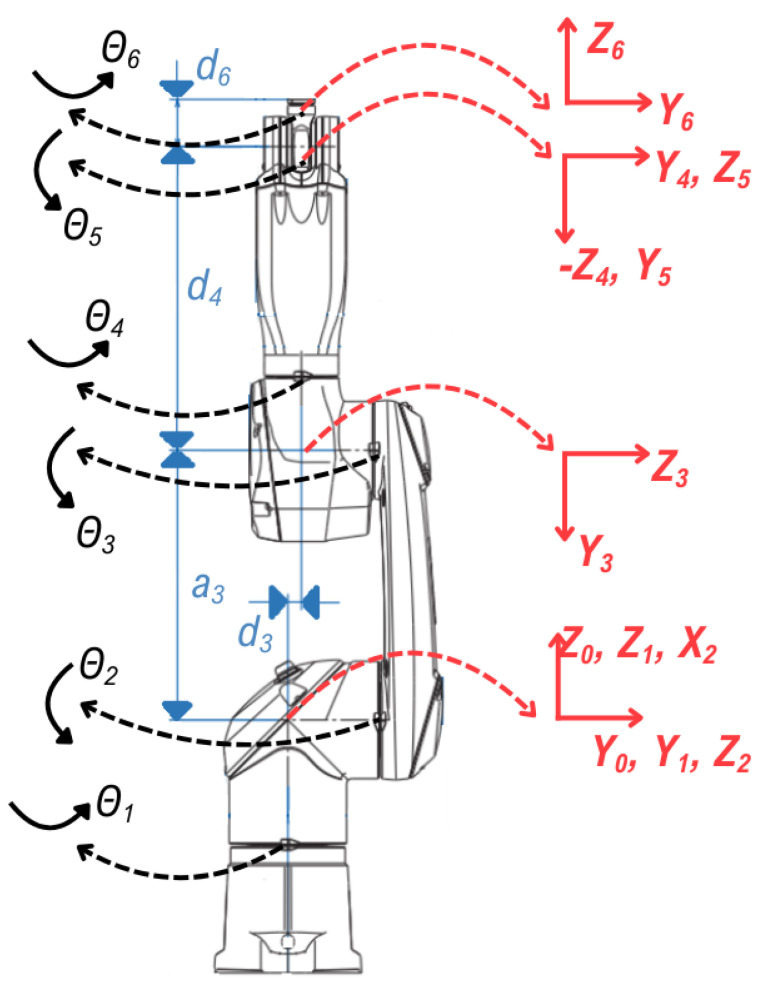
The link frame assignments of the Staubli TX2-60L.

In this study, forward kinematic calculations are performed to find the position and orientation of the end-effector frame {T} of a 6-DOF Staubli robot arm in Cartesian space with respect to the base frame {B}. A standard Denavit–Hartenberg (DH) method [47] is used to create the kinematic model of the robot and to perform forward kinematic calculations. This method defines the geometric relationship between each joint and link with four DH parameters (α, *a*, *d*, and θ). According to the link frame assignments of the Staubli TX2-60L robot arm we created, as shown in Figure 3, the DH parameters for each link are determined as in Table 1.

In Table 1, $\alpha_{i}$ is the twist angle between two joint axes, $a_{i}$ is the distance between two adjacent joint axes, $d_{i}$ is the translation from one link to the other along the joint axis, and $\theta_{i}$ is the angle of rotation about the joint axis.

The variable DH parameters of this robot arm, all six of which are rotary joints, are the joint angles $\theta_{i}$ for each joint and are measured from the encoder data in the motors/actuators. The other three DH parameters of the robot arm ($\alpha_{i}$, $a_{i}$, and $d_{i}$) are fixed. These constant parameters are $a_{3}=0.4$ m, $d_{3}=0.02$ m, $d_{4}=0.45$ m, and $d_{6}=0.07$ m. Based on the standard definitions of the DH parameters [48], link-to-link transformation matrices are formed as

(1) $$T_{i}^{i-1}=\left[\begin{array}{cccc} c(\theta_{i}) & -c\left(\alpha_{i}\right)s\left(\theta_{i}\right) & s\left(\alpha_{i}\right)s\left(\theta_{i}\right) & a_{i}c\left(\theta_{i}\right)\\ s(\theta_{i}) & c\left(\alpha_{i}\right)c\left(\theta_{i}\right) & -s\left(\alpha_{i}\right)c\left(\theta_{i}\right) & a_{i}s\left(\theta_{i}\right)\\ 0 & s(\alpha_{i}) & c(\alpha_{i}) & d_{i}\\ 0 & 0 & 0 & 1 \end{array}\right]$$

where $s(\theta_{i})$ and $c(\theta_{i})$ represent the sine and cosine of the joint angle $\theta_{i}$, respectively, and $s(\alpha_{i})$ and $c(\alpha_{i})$ represent the sine and cosine of the twist angle $\alpha_{i}$, respectively.

When each link-to-link transformation matrix is calculated from $T_{1}^{0}$ to $T_{6}^{5}$ and multiplied by all, the transformation matrix that gives the pose information of the end-effector of the robot arm $T_{6}^{0}$ with respect to the base is found as

(2) $$T_{6}^{0}=T_{1}^{0}T_{2}^{1}T_{3}^{2}T_{4}^{3}T_{5}^{4}T_{6}^{5}=\left[\begin{array}{cccc} r_{11} & r_{12} & r_{13} & p_{x}\\ r_{21} & r_{22} & r_{23} & p_{y}\\ r_{31} & r_{32} & r_{33} & p_{z}\\ 0 & 0 & 0 & 1 \end{array}\right]$$

The equations for the 3×1 position vector entries ($p_{x}$, $p_{y}$, and $p_{z}$) obtained from the base-to-end-effector transformation matrix in (2) can be calculated according to the DH parameters as

(3) $$p_{x}=d_{4}s_{23}c_{1}-d_{3}s_{1}-d_{6}\left(s_{1}s_{4}s_{5}-s_{23}c_{1}c_{5}-c_{1}c_{2}c_{3}c_{4}s_{5}+c_{1}c_{4}s_{2}s_{3}s_{5}\right)+a_{2}c_{1}c_{2}$$

(4) $$p_{y}=d_{6}\left(c_{1}s_{4}s_{5}+s_{23}c_{5}s_{1}+c_{2}c_{3}c_{4}s_{1}s_{5}-c_{4}s_{1}s_{2}s_{3}s_{5}\right)+d_{3}c_{1}+d_{4}s_{23}s_{1}+a_{2}c_{2}s_{1}$$

(5) $$p_{z}=d_{4}c_{23}-a_{2}s_{2}-0.5\left(d_{6}s_{23}s_{45}\right)+d_{6}c_{23}c_{5}+0.5\left(d_{6}\sin\left(\theta_{4}-\theta_{5}\right)s_{23}\right)$$

Using the 3 × 3 rotation matrix of the robot arm obtained from the base-to-end-effector transformation matrix in (2), the Euler angles (roll $r_{x}$, pitch $r_{y}$, and yaw $r_{z}$) representing the orientation of the end-effector in Cartesian space can be calculated according to the DH parameters as

(6) $$r_{x}=\arctan\left(c_{23}s_{5}+s_{23}c_{4}c_{5}+s_{23}c_{6}s_{4},\;c_{23}c_{5}-s_{23}c_{4}s_{5}\right)$$

(7) $$r_{y}=\arctan\left(c_{6}\left(c_{23}s_{5}+s_{23}c_{4}c_{5}\right)-s_{23}s_{4}s_{6},\sqrt{r_{11}^{2}+r_{21}^{2}}\right)$$

(8) $$\begin{array}{ccc} r_{z} & = & \arctan(s_{6}\left(c_{1}c_{4}-c_{2}c_{3}s_{1}s_{4}+s_{1}s_{2}s_{3}s_{4}\right)-c_{6}\left(s_{23}s_{1}s_{5}-c_{1}c_{5}s_{4}-c_{2}c_{3}c_{4}c_{5}s_{1}+c_{4}c_{5}s_{1}s_{2}s_{3}\right)\\ & & -s_{6}\left(c_{4}s_{1}+c_{1}c_{2}c_{3}s_{4}-c_{1}s_{2}s_{3}s_{4}\right)-c_{6}\left(c_{5}s_{1}s_{4}+s_{23}c_{1}s_{5}-c_{1}c_{2}c_{3}c_{4}c_{5}+c_{1}c_{4}c_{5}s_{2}s_{3}\right) \end{array}$$

where $r_{11}=-s_{6}\left(c_{4}s_{1}+c_{2}c_{3}s_{4}-c_{1}s_{2}s_{3}s_{4}\right)-c_{6}\left(c_{5}s_{1}s_{4}+s_{23}c_{1}s_{5}-c_{1}c_{2}c_{3}c_{4}c_{5}+c_{1}c_{4}c_{5}s_{2}s_{3}\right)$ and $r_{21}=s_{6}\left(c_{1}c_{4}-c_{2}c_{3}s_{1}s_{4}+s_{1}s_{2}s_{3}s_{4}\right)-c_{6}\left(s_{23}s_{1}s_{5}-c_{1}c_{5}s_{4}-c_{2}c_{3}c_{4}c_{5}s_{1}+c_{4}c_{5}s_{1}s_{2}s_{3}\right)$.

## 3. Industrial Camera Setup

**Figure 4:**
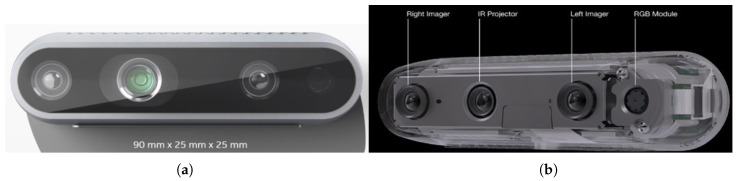
Intel RealSense camera views: (**a**) Outer view. (**b**) Inner view.

The Intel RealSense D435 camera (as shown in Figure 4) is a dual-lens stereo-based RGB–depth-type camera and provides depth perception in various industrial applications. It has a 1920 × 1080 frame resolution, 30 fps frame rate, and 69 × 42 degree field of view with the RGB sensor and a 1280 × 720 output resolution, 90 fps frame rate, and 87 × 58 degree field of view with the stereoscopic depth sensor. It has an ideal working distance of 0.3 m to 3 m. All of these features are the reason why we prefer to use it in our robotic applications. In our setup, we fixed our camera 750 mm away from the object table frame. The connection between the camera and the PC is provided by USB 3.1 communication.

### 3.1. Calibration of Camera and Robot Frames

The calibration of the camera and the robot allows the transformation of the image coordinates (object frame {O}) into world coordinates (camera frame {C}, robot tool frame {T}, and robot base frame {B}). By projecting the camera frame {C} onto the robot tool frame {T}, the robot can operate directly within its own coordinate system (robot base frame {B}), using orientation and position data. A precisely calibrated system is essential in this field. These enhanced results help improve the accuracy and repeatability of the robot arm. Various calibration techniques are available depending on the specific requirements of robotic applications [49]. For our purposes, we used the calibration plate method [50].

**Figure 5:**
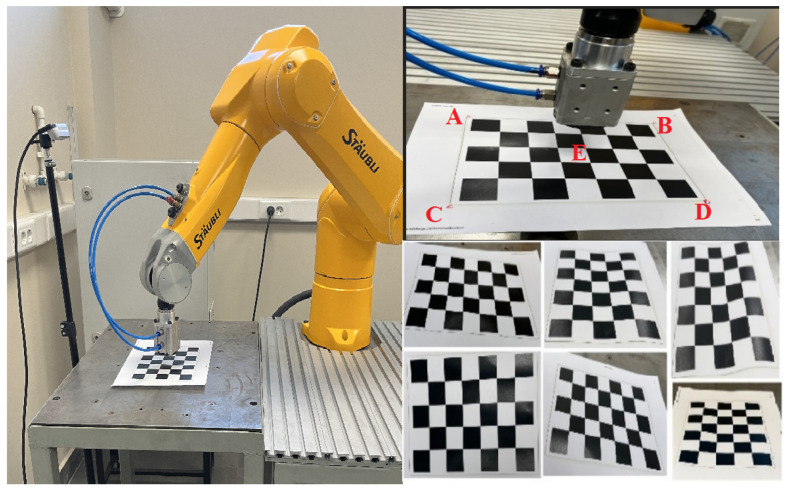
Overall calibration system structure.

In this calibration method, the absolute positions on the robot tool frame {T} are determined by means of a calibration plate. To achieve this, we need to take several images of the calibration plate and four corner reference marks to be noted. With the help of a measuring tape (with the measurement of the joint positions and the knowledge of the forward kinematics of the robot), we can determine the position of the calibration plate relative to the robot tool frame {T}. This calibration process gives the output of the image coordinates (object frame {O}) obtained from the camera. Figure 5 shows the calibration processes for the calibration plate and the robot’s end-effector.

The calibration plate is mounted on a table with dimensions of 650 × 450 mm and contains 7 × 5 square cells of 25 × 25 mm each. The distance between the origin of the camera frame and the origin of the calibration plate frame is 750 mm. The camera resolution is selected as 1280 × 720. The maximum resolution value of the X-axis is 1280 and the maximum resolution value of the Y-axis is 720. The camera captures the image and defines the four end-point pixel values A, B, C, D, and E on the calibration plate, as shown in the upper right of Figure 5.

After the calibration process, we can formulate the position of a point (the center of the object) obtained from the camera image on the X- and Y-axes relative to the robot’s tool frame as follows:

(9) PxOT=(Xmax−Xact)Xcon

where $X_{max}$ is the maximum resolution value (1280 pixels) of the X-axis in the camera frame {C}, $X_{act}$ is the measured resolution value of the X-axis in the camera frame {C}, and $X_{con}$ is the conversion ratio (0.0565) of the X-axis between the camera frame {C} and the robot tool frame {T} when the robot arm is in the home position.

(10) PyOT=(Ymax−Yact)Ycon

where $Y_{max}$ is the maximum resolution value (720 pixls) of the Y-axis in the camera frame {C}, $Y_{act}$ is the measured resolution value of the Y-axis in the camera frame {C}, and $Y_{con}$ is the conversion ratio (0.064) of the Y-axis between the camera frame {C} and the robot tool frame {T} when the robot arm is in the home position.

To complete the calibration process, the values of pixels on the X- and Y-axes are converted to mm according to the calibration plate information. These coordinates from the camera need to be transformed into the coordinates of the robot by using the rotation matrix in (2).

In practice, the calibration between the robot and the camera frames remains valid as long as the physical positions of the camera and the robot base are unchanged. Therefore, recalibration is not required during routine operation, even if the positions of the objects on the production line vary. The calibration procedure itself is simple, involving the use of a calibration plate with the setup, and typically takes only a few minutes to complete. This makes the system suitable for long-term deployment in stable production environments.

### 3.2. Image Acquisition Using LabVIEW

In order to interface with the robotics and camera setup in industrial applications, a robust software infrastructure is needed to acquire and process image data. In this study, we used LabVIEW from National Instruments (NI) to access and acquire camera data and also used the NI Vision library, which will be described in the next section, to process the data from the acquired image. LabVIEW is a graphical programming language. This program provides a good solution that is compatible with our existing industrial automation infrastructure for real-time processes and also provides an easy connection to any device or interface. All the software infrastructure for interfacing with real-world signals, analyzing data, and driving our robot control system was developed on LabVIEW.

**Figure 6:**
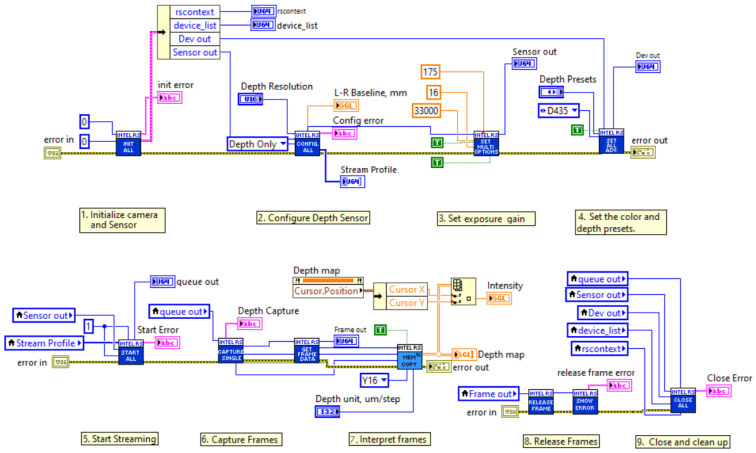
The developed LabVIEW-based image acquisition standalone software.

An independent software with real-time processing was developed to obtain images from an industrial camera using the NI LabVIEW program. The LabVIEW-based image acquisition software shown in Figure 6 consists of nine different formation blocks and runs on a personal computer (PC) with a Windows 10 operating system (OS), connected to the camera via a USB3.1 port. The images acquired with LabVIEW software are then processed with LabVIEW’s NI Vision library. The camera image is first converted to a 2D rectangular form and then sent to processing. The depth resolution of the image is in RGB format, with each pixel representing 8-bit red, green, and blue color values. The developed LabVIEW-based image acquisition standalone software is explained in detail in the following.

#### 3.2.1. Camera Initialization

In this initialization section, it is necessary to provide details of the camera features and the sensors used. Depending on the application requirements, the Intel RealSense camera used may have various features and sensors. More than one camera device can be connected to a PC and each camera may have more than one sensor. For example, the D435 model camera has a stereo sensor and an additional RGB sensor. Other types of cameras may also have different types of sensors, such as an external IMU. Each RGB sensor may have more than one stream, and each stream has a profile. For example, these profiles may have frame rates, resolution, format, etc. Therefore, we need to initialize the camera with the appropriate configuration according to our requirements. In our LabVIEW-based software, the index is specified as ‘0’ to initialize our camera with one RGB sensor and one stream profile at the beginning.

#### 3.2.2. Depth Sensor Configuration

As shown in Figure 6, the depth sensor configuration is completed in the second stage of our LabVIEW software. This stage is important for reading the density of the object. The image density from the camera depth sensor is used to measure the Z-position of the points on the image surface with respect to the camera frame. The Z-axis of the camera frame is calculated according to the distance from the camera to the object whose image is taken according to the density value. In our setup, the camera depth sensor has a 90 fps frame rate and 87 × 58 degree field of view.

#### 3.2.3. Exposure Gain

This stage is used to adjust the gain of the laser sensor to obtain better performance from the depth sensor. The depth sensor has an auto-exposure function and automatically adjusts the gain and exposure. In this study, we usually used low values; for example 16 fps for the frame rate and 300 mW for the laser power were found to be sufficient.

#### 3.2.4. Setting Presets

At this stage of the software, the presets used for the depth and color settings are coded. According to our configuration, the D435 camera has an RGB sensor and measures depth using intensity. Different presets are derived for depth measurement with different exposure gains and laser power values. The depth of the image, and therefore the intensity, can be affected by various environmental factors (light, reflection, dust, etc.), low-level sensor values, or image overlap, among many other factors.

#### 3.2.5. Start Streaming

After the configuration of the camera and the image sensors, at this stage we start the process of streaming the acquired images from the camera continuously and in real time. Here, it is necessary to give an input for the capacity and number of images according to the camera and sensor connected to the system. In the developed software, a camera is connected to a sensor. Therefore, the capacity and stream count are set to ‘1’, as seen in Figure 6.

#### 3.2.6. Capturing and Process

This system captures images from the configured camera and the connected sensor. The image details are outputted at this stage with height, width, frame details, camera format, and image data types.

#### 3.2.7. Interpret and Display Frames

The interpretation of image processing frames is primarily based on the frame capture process. The images used are marked as images containing spatial depth information in the X- and Y-axes and encoded in the Y16 grayscale format. This Y16 image represents a 16-bit grayscale format where each pixel represents the ‘Y-luminance’ component of a ‘YCbCr’ color model. These images contain intensity values in the form of a 2D array. The intensity component of the processed image is extracted according to the selected reference image by developing it with the filter designed in the LabVIEW environment. The results obtained are presented visually in both ‘depth image’ and ‘color image’ formats. In addition, the final processed image is archived in the U16 (16-bit unsigned) grayscale format for future verification after the process is stopped. This converted U16 format represents pure intensity values of the image.

#### 3.2.8. Release Frames

The next image capture process is to release the captured frames from the camera and the depth sensor. The memory and buffer are cleared for the next capture. The only input in the memory will be the details of the captured frame.

#### 3.2.9. Close Function

In this final stage of the image acquisition software, the entire camera and sensor configuration is closed. The frames in the buffer are also closed to safely exit the process.

The stages outlined above in our LabVIEW-based software are used to capture images from the camera. The settings and configuration of the software are selected according to the fixed position of our camera for the image plane and our setup. The selected settings and configurations may change when we change the setup. It is important to emphasize that all the experimental studies described in the following were always performed with the same setup and under the same physical conditions. After capturing the image, it is sent to the NI vision system, which will be described in the next section, to find the correct object from the image, calculate its coordinates, and measure its dimensions.

## 4. Image Processing with Analytics Solution Using NI Vision

The working concept of an image-guided robotics system (machine vision) is straightforward. A picture of an object is taken from an image sensor (camera), and a PC analyzes the image to send the coordinates of the object to a robot arm so it can move to the desired position. In this section, we explain the image processing techniques and our analytical solution approach with the software developed using LabVIEW’s NI Vision library to measure the size, frame, and pose of the objects (metal plates) from the images taken from the LabVIEW-based software. In our analytical solution approach, we find all the pxOC, pyOC, and pzOC positions and rxOC, rxOC, and ryOC rotations of the object frame {O} in the Cartesian space, in the six axes, with respect to the camera frame {C}. The NI Vision library is used in the LabVIEW software to find the pzOC and pyOC positions of the object and the rzOC angle with our software algorithm. The position pzOC is calculated by matching the depth camera intensity. Trigonometric inclination angle calculations are computed analytically in our software algorithm to find the rotations rxOC and ryOC. All of these techniques are explained in detail below.

### 4.1. Finding pxOC, pyOC, rzOC Using NI Vision

**Figure 7:**
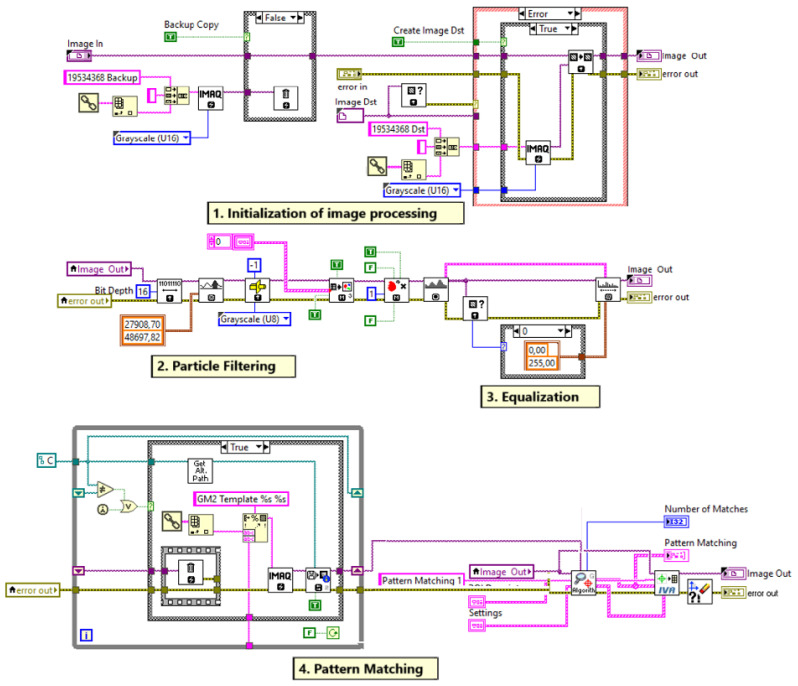
The developed NI Vision-based image processing standalone software.

In the process of finding the coordinates of the object, we use the NI Vision library with the software algorithm we developed to find pxOC, pyOC, and rzOC. In this process, the reference image of the object to be found and selected is given to the algorithm. Then it is necessary to process the image taken from the camera. There are various intermediate methods in these image processing techniques. These are filtering, the histogram technique, image enhancement, particle analysis, and pattern matching to find the NI Vision tool code, as shown in Figure 7.

#### 4.1.1. Initialization of Image Processing

After successful image acquisition, the captured image must be converted to the required formats (binary, grayscale, and RGB). In binary format, the values of the image pixel are 0 or 1. In grayscale format, image pixels take values in the range of 0–255 or 0–65,535 according to 8- or 16-bit values. After these formatting processes, pattern matching techniques are applied in image analysis and processing methods in NI Vision. The methods that must be performed for pattern matching are thresholding, particle filtering, and histogram equalization. Then this output is used in the pattern matching process for object detection.

**Figure 8:**
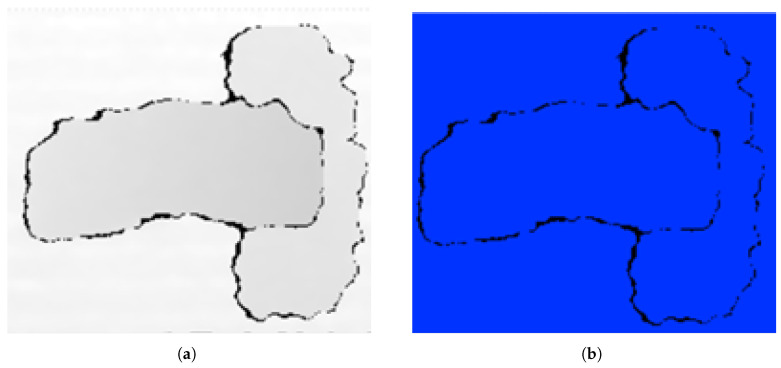
The threshold stage consists of (**a**) grayscale input image and (**b**) grayscale output image.

As shown in the upper part of Figure 7, in the NI Vision-based software, the threshold system consists of an image bit depth configuration and an image threshold configuration. The threshold is used to obtain grayscale objects, as shown in Figure 8. An unsigned 16-bit grayscale image is used from the captured and/or recorded image. Before the image enters the thresholding process, a 16-bit depth modification must be applied to the image. Then, values are entered for alignment of the system, camera position, and distance adjustment in the image thresholding process. Low values such as 26,617.39 and high values such as 65,500.00 can be selected. The threshold values of the 16-bit image can be changed between 0 and 65,535.

#### 4.1.2. Particle Filtering

**Figure 9:**
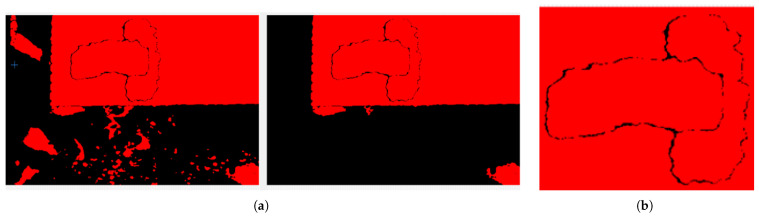
Particle filtering stage consists of (**a**) particle filter input and output images and (**b**) particle-filtered image.

The particle filtering technique at the second stage of the NI Vision software developed, as shown in Figure 7, is used in image processing processes to distinguish objects with certain characteristics (such as the unique shape of the brake pad metal plate in this study). This method eliminates unwanted components in the image through filtering processes applied at the neighborhood level. During the filtering process, each particle is evaluated on the basis of criteria such as height, width, and mass (pixel density). When the image is captured, the target object of interest is focused on. However, there may be many unwanted details in this image that are not analyzed. Particle filtering eliminates these unwanted regions. Although it is not possible to completely eliminate all unwanted elements, a partially cleaned grayscale image is obtained as a result of the method shown in Figure 9. This intermediate output provides a suitable basis for more complex analyses to be performed in the following stages. Filtering results vary depending on the specified pixel size ranges (e.g., width, height, and mass limits). Thus, particles that do not meet certain criteria are removed from the image, creating a simpler and more processable image.

#### 4.1.3. Equalization

**Figure 10:**
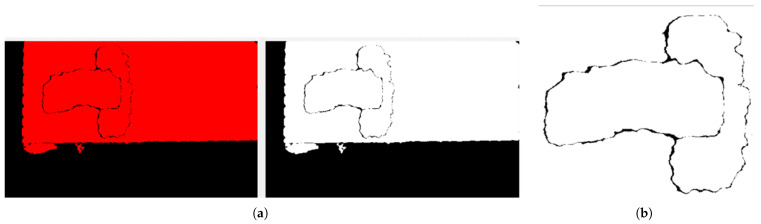
Equalization stage consists of (**a**) histogram input and output images and (**b**) equalized image.

In the equalization stage, which is the third step of the NI Vision software developed, as shown in Figure 7, the filtered image is converted to a grayscale image in 8-bit format. That is, the value 0 represents black and the value 255 represents white. The preprocessed grayscale image has only 0 and 1 representation. In the histogram equalization stage, the contrast of the grayscale image is increased and an evenly distributed output image is obtained. This equalization stage is used to increase the pattern of the object as much as possible and makes the pattern of the object more distinct, as shown in Figure 10.

#### 4.1.4. Pattern Matching

**Figure 11:**
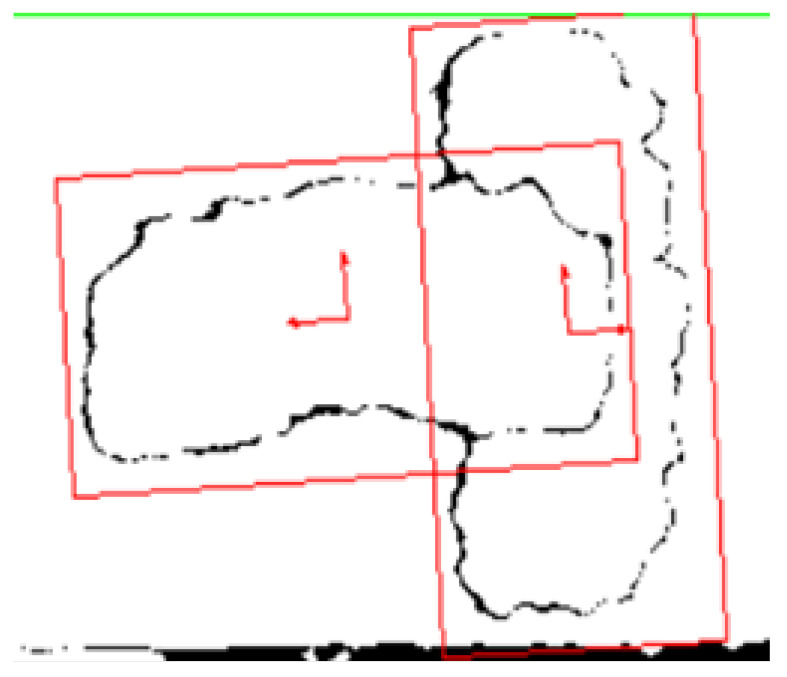
The pattern matching system’s output image.

The pattern matching technique in the final stage of the NI Vision software developed, as shown in Figure 7, is used to detect the shape of the image based on the reference image. Scaled, implicit, and rotated parameters are used as a configuration to obtain the pattern matching of the object. First, the maximum pattern range that we can obtain according to the scaled values is given. Then, the visual field of the object is structured, and the total pattern-matched object is obtained. Then, by applying the edge detection technique, a calculation is made to obtain the position values pxOC and pyOC. For this calculation, the start and end values of the object are taken in accordance with the pixel values. According to the results, the center point of the coordinate values of the image that has now been pattern-matched is calculated. This center point gives the pxOC and pyOC positions of the object according to the camera frame {C}. The patterned object is compared with the reference object to calculate the rzOC angle of the object and the rotation angle in the Z-axis in the 2D plane is calculated. As a result, we obtain the pxOC and pyOC positions of the center coordinates of the object and the rotation angle rzOC in the Z-axis using the pattern matching technique. The output of the pattern matching stage is shown in Figure 11.

With this software we developed based on NI Vision, we calculate the positions pxOC and pyOC and the rotation angle rzOC of the center point of the object according to the reference image.

### 4.2. Finding pzOC Using Camera Intensity

**Figure 12:**
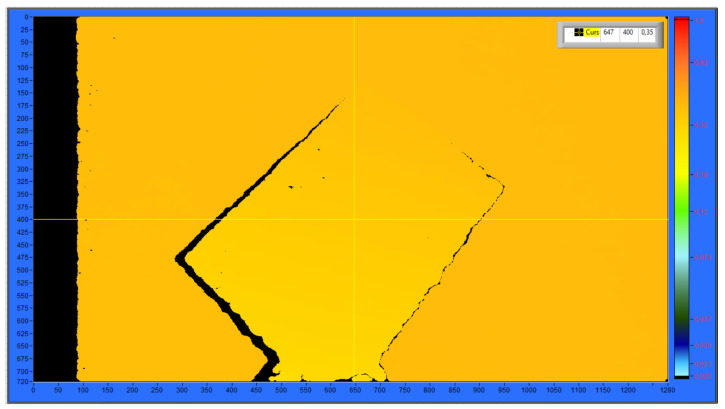
Camera intensity value display.

In this section, the pzOC position of the center point of the object is calculated from the intensity values with the help of the depth-sensing feature of the Intel RealSense camera. The different intensity values from the focal point of the camera to the object vary between ‘0’ and ‘1’. These intensity values also represent the color change. For example, the lowest intensity value of ‘0’ represents the color white, and the highest intensity value of ‘1’ represents the color red. An example representation of the intensity pattern values can be seen in Figure 12.

The intensity values detected at the center point of the object obtained in the previous stage are converted to the distance in mm. This distance gives the pzOC position of the center point of the object relative to the camera frame {C}.

### 4.3. Finding rxOC and ryOC Using Angle-of-Inclination Analytic Method

In this section, we find the geometrical rxOC and ryOC rotation angles from the poses of the object in Cartesian space using the angle-of-inclination technique. In this technique, inclination angle calculations are performed with trigonometric concepts. The inclination angle formulation can be defined as follows, where a line of the object in the horizontal position is in the 2D plane with respect to the camera’s X and Y coordinates.

(11) $$\phi =\tan^{-1}\left(\frac{\Delta Y}{\Delta X}\right)$$

where ϕ is the angle the line makes with the positive X-axis, ΔY is the difference $Y_{2}-Y_{1}$ between the starting $Y_{1}$ and ending $Y_{2}$ coordinate points on the Y-axis of the line, and ΔX is the difference $X_{2}-X_{1}$ between the starting $X_{1}$ and ending $X_{2}$ coordinate points on the X-axis of the line. The inclination angle of the object’s horizontal line relative to the camera’s X-axis gives the rotation angle ryOC, as in (11). In contrast, the inclination angle of the object’s horizontal line relative to the camera’s Y-axis gives the rotation angle rxOC, as given below.

(12) $$\phi =\tan^{-1}\left(\frac{\Delta X}{\Delta Y}\right)$$

**Figure 13:**
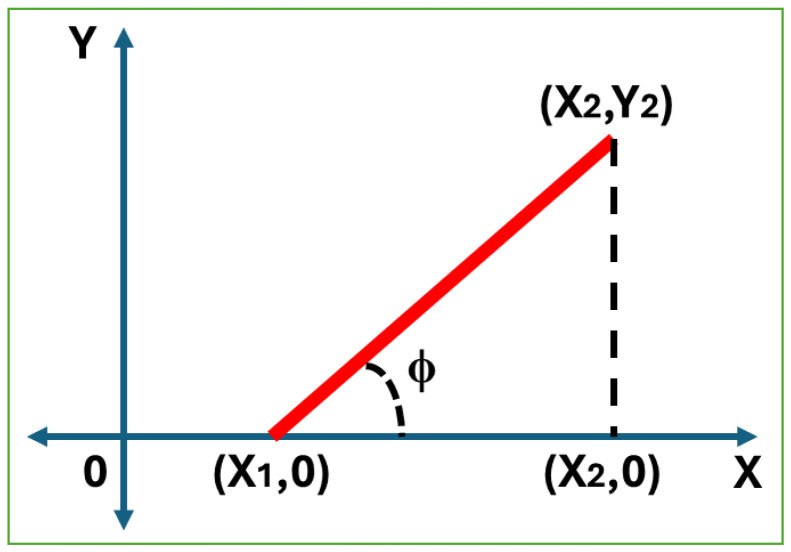
Calculation of the inclination angle (ϕ) of an object line.

An example of an inclination angle calculation from our experimental study can be seen in Figure 13. Here, when the first point of an object line with a positive slope is $\left(X_{1},0\right)$ and the second point is $\left(X_{2},Y_{2}\right)$ on the X-Y plane, we can find the angle of inclination as $\phi =\tan^{-1}\left(\frac{Y_{2}}{X_{2}-X_{1}}\right)$.

**Figure 14:**
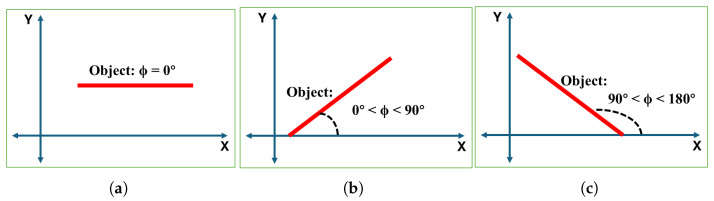
Inclination angle (ϕ) of the line (object) representation: (**a**) horizontal inclination, (**b**) positively sloped inclination, and (**c**) negatively sloped inclination.

As seen in Figure 14, the angle of inclination of a horizontal line is always 0°. The inclination of a non-horizontal line is ϕ, which is always considered to be within 0°<ϕ<180°, measured counterclockwise from the X-axis to the line.

Using the angle-of-inclination technique and the intensity values coming from the camera, we find the $r_{x}$ and $r_{y}$ rotation angles. If the object has an inclination relative to the plane of the X-axis, we must first find the $r_{y}$ rotation angle. This angle of inclination and angle plane are derived from the change in the camera’s intensity values. To find the inclination angle of the object, we need to confirm how the object is placed on the plane (with $r_{x}$ or $r_{y}$ rotation). The edge detection technique is applied to find the location of the object on the plane surface. Using this edge detection technique, the starting and ending pixel values of the object can be found. The pixel values of the object image on the X-axis are incremented from the center point to the starting and ending pixel values. A similar operation must be performed for the pixel values on the Y-axis. With these pixel increments, the length of the object and also the rotations at which $r_{x}$ or $r_{y}$ it is placed on the axis are calculated. That is, if the increment in the pixels on the X-axis successfully reaches the starting or ending pixel values of the object, then it can be verified that the object to be picked has a rotation angle of $r_{y}$. Similarly, the rotation angle of $r_{x}$ is verified using the increment in the pixel values on the Y-axis. For example, after the object’s edge detection is performed, the starting point of the determined object line is intersected on the X-axis, and the inclination angle is calculated on the X-axis plane. In other words, if there is a height difference between two edges, there is a clear change in the intensity of the image. In this case, it is concluded that there is an angular rotation between the two edges. Then, it is found which of the object line coordinate points belong to the X-axis or Y-axis plane. Then, it is concluded that this object has $r_{x}$ or $r_{y}$ rotation angles.

## 5. Image Processing with Hybrid-Based Methods

Based on the comprehensive review of the literature presented in Section 1, the methods most commonly used in recent studies on 6D pose estimation include PnP, RANSAC, YOLO, and various hybrid algorithmic approaches that combine these techniques. Therefore, in this study, we will compare our analytical solution-based 6D pose estimation method with hybrid-based approaches YOLO + PnP/RANSAC. In hybrid-based approaches, while the key points of the object are determined with YOLO as a deep learning-based object detector, the 6D pose of the object is calculated with PnP/RANSAC as classical geometric-based solution methods.

### 5.1. Detecting Object Key Points Using YOLO-v8

In this study, as the first and most important step in determining the 3D position of the metal plates using methods based on deep learning, we use a YOLO-based pose estimation model, which is one of the most widely used methods in this field in the literature, to accurately and reliably detect key points in the 2D image. The YOLO model detects five key points (corners and a reference point) on the metal plate with high accuracy and speed. The reference point plays a critical key role in the 3D position estimation of the object regardless of the visual orientation of the metal plate. The 2D coordinates of the key points detected by YOLO are used for the 6D pose estimation of the metal plate in the following sections.

In order to accurately detect the key points of the object, the YOLO model goes through a comprehensive four-stage training process. Firstly, in the dataset preparation stage, we use 2000 specially labeled plate images for model training. In each of these images, five key points of the metal plate are automatically marked via the Roboflow SDK in Python (ver.3.15) code. Secondly, in the training configuration stage, the model is trained with a batch size of four for 100 epochs. A dynamic learning rate between $3.3233\times 10^{-5}$ and $8.911\times 10^{-5}$ is used to ensure that the model both gets a quick start and makes finer adjustments as training progresses. Third, in the performance metrics stage, the final value of ‘train loss’ obtained at the end of training is 0.2564. This low value shows that the model exhibits high precision in key point estimations. In addition, mAP50(B) and mAP50-95(B), which are mean average precision metrics (mAP) showing the generalization ability of the model, are recorded as 0.9125 and 0.91472, respectively. These high mAP values confirm that our model detects the metal plate points and bounding boxes with high accuracy and reliability. Finally, in the stage of prevention of overfitting, an early stopping mechanism is integrated to prevent the model from memorizing only the training data and to ensure that it performs well on new images. In this way, the model is automatically stopped at epoch 76 instead of epoch 100, which prevents the model from running the risk of overfitting and losing its generalization ability. Although the YOLO-v8 model used in this study was trained on 2000 manually labeled images of brake pad plates, applying the same approach to different object types would require additional annotation effort.

### 5.2. Camera Calibration and Correcting Image Distortions Using OpenCV

Before transferring the results of model training obtained with YOLO to the pose estimation stage with PnP methods, camera calibration is first performed to make accurate measurements and correct 3D inferences. The Intel RealSense camera that we use is calibrated in the software that we developed with Python code using the open-source OpenCV library. With this calibration process, two basic features of the camera are learned. One is the internal parameters of the camera, which are the focal length of the camera in pixels and the coordinates of its optical center. The other is the radial and tangential distortion coefficients, which cause various distortions in the image. Then, using the internal parameters and distortion coefficients obtained, the undistorted position of each pixel in the distorted (original) image is calculated and moved to these new positions, thus obtaining a geometrically clearer image.

### 5.3. Six-Degrees-of-Freedom Pose Estimation Using PnP/RANSAC Algorithms

After detecting the key points of the object using YOLO-v8 and editing the camera images using OpenCV, we now try to find the 6D pose of the object using PnP, RANSAC, and their hybrid algorithms as classical geometric-based solution methods.

The main purpose of PnP algorithms is to calculate the 6D pose of the camera relative to the object as a rotation matrix and a translation vector using the geometric relationship between the projections of several points of the object whose 3D coordinates we know (five key points, four corners, and one reference for the metal plate used in this study) on the 2D image of the camera. In the literature [13], various PnP algorithms have been developed for different situations and needs. Some methods that are considered appropriate for the purpose of this study are derived from the most widely used PnP-based algorithms in the literature.

#### 5.3.1. Solving PnP with EPnP

In the efficient perspective-n-point method (EPnP) [51], all 3D points of the object are defined as a weighted linear combination of four virtual control points (barycentric coordinates). A linear equation based on the control points is obtained using 2D projection equations. The rotation matrix and translation vector are calculated from the corresponding control points in the object and camera frames. The EPnP method solves the PnP problem quickly and easily.

#### 5.3.2. Solving PnP with IPPE

The infinitesimal plane-based pose estimation method (IPPE) calculates the homography matrix, which defines the projective transformation between two planes (the 3D plane where the object is located and the 2D image plane of the camera) [52]. From this matrix, which contains the internal and external parameters of the camera, the rotation matrix and the translation vector are extracted with special mathematical decomposition techniques. This method is ideal for planar plates (such as the metal plates used in this study).

#### 5.3.3. Solving PnP Iteratively

The Iterative PnP method, first introduced by [53], starts with a possible pose of the camera relative to the object and iteratively updates the camera pose using a non-linear optimization to minimize the projection error obtained by projecting the 3D points onto the 2D camera image plane using the current estimated pose. This optimization uses an optimization technique such as Levenberg–Marquardt to analyze how the error varies with the pose parameters and update these parameters in small amounts to reduce the error. This process is continued until the error falls below a certain threshold value, the change in the parameters becomes very small, or a certain maximum number of iterations is reached. This method generally provides higher accuracy but is slower than other methods.

#### 5.3.4. Solving PnP with AP3P

This method uses geometric calculations between three known 3D points on the object and their projections on the camera image [54]. These geometric calculations include both the known distances between the 3D points and the angles in the triangles formed by these 3D point pairs with the camera’s optical center. These angles can be calculated using the 2D points in the image and the camera’s intrinsic parameters. With this information, an equation structure is established for each point–optical center triangle by applying the cosine theorem to the unknown distances from the camera to the 3D points. Then these equations are algebraically manipulated into a fourth-degree polynomial equation with a single variable. The roots of this polynomial give possible solutions for the distances and can therefore yield up to four different pose estimates. Additional checks are made for the correct solution (e.g., using a fourth point). This method is a type of PnP that can perform 6D pose estimation analytically with high accuracy.

#### 5.3.5. Solving PnP with RANSAC and Hybrid Approaches

Although PnP algorithms work very well with correct data, in practice, there may be some false matches when finding 2D counterparts of 3D points in the image (e.g., when marking the corners of a metal plate or performing automatic matching). These false matches, namely outliers, can seriously distort the PnP result. In this case, a useful method, such as RANSAC, can be used to eliminate these false or irrelevant data and base the PnP solution only on the correct matches (inliers) that fit our model.

RANSAC is not a PnP algorithm itself but rather a framework or wrapping method that makes the above-mentioned PnP algorithms (EPnP, IPPE, Iterative, and AP3P) more resilient to erroneous data [39]. RANSAC+PnP methods, RANSAC/EPNP, RANSAC/P3P, RANSAC/IPPE, and RANSAC/ITERATIVE hybrid approaches, generate multiple PnP solutions with randomly sampled point matches and try to find robust pose estimation against outliers by selecting the one that provides the most inliers. In this way, the probability of PnP algorithms making large errors due to incorrect point matches is significantly reduced, and much more reliable pose estimations are obtained.

## 6. Experimental Studies

**Figure 15:**
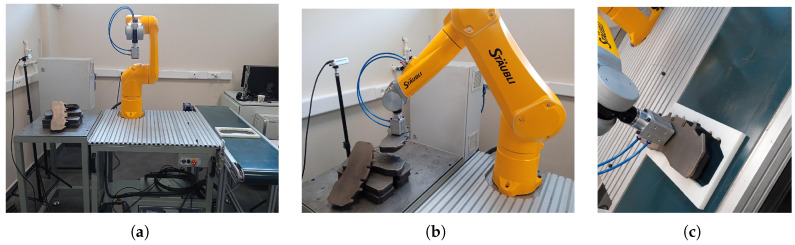
Real experimental study setup: (**a**) Robot arm at home position. (**b**) Robot arm at picking position. (**c**) Robot arm at placing position.

The experimental setup consists of an industrial robot arm and a camera for real pick-and-place industrial applications. In the setup, we will test both our own analytical solution software algorithm and hybrid algorithms (YOLO+PnP/RANSAC) to estimate the 6D pose of the metal plates. As seen on the left in Figure 15, in our experimental setup, the robot arm first moves from the home pose to the object picking pose. The target pose for this movement in Cartesian space is estimated from camera images with analytical solution or hybrid-based methods. As seen in the middle of Figure 15, the robot arm should hold the metal plate with the vacuum–magnetic gripper in the correct pose so that it can place the metal plate in the white-colored empty slot with a known pose with full precision, as seen on the right of Figure 15. The empty slot used in the placement process here has an offset of 2 mm wider than the X-Y dimensions of the metal plates.

### 6.1. Pick-and-Place Application Software

**Figure 16:**
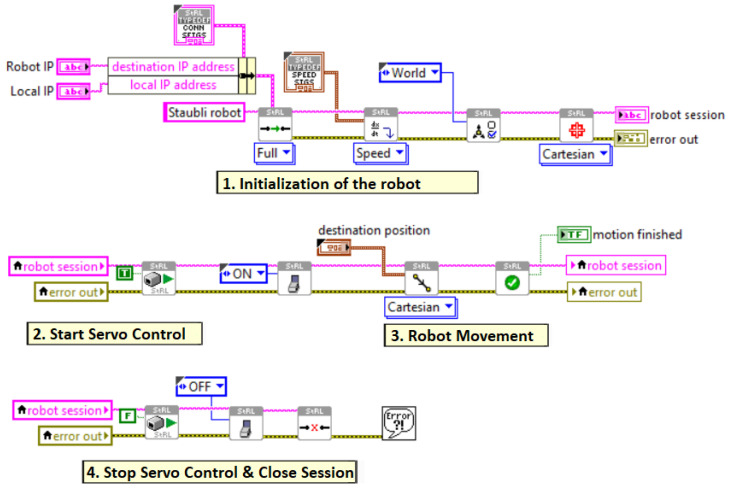
LabVIEW-based control software for the robot arm.

In pick-and-place applications of the Staubli robot arm, LabVIEW-based software is developed for the CS9 controller for movement control of the robot arm. Figure 16 shows the functional diagram of the LabVIEW-based pick-and-place application software for the robot arm.

The LabVIEW-based robot control software includes four stages, as can be seen in Figure 16. In the first initialization stage, the robot and system port identification numbers and IP addresses are entered to establish a connection with the PC and the robot arm’s CS9 controller. Then, the speed limits of the servo motors in the joints of the robot arm are defined. For the initial setup, the maximum speed of the servo motors is set to 250 mm/s. In addition, the Cartesian space coordinate data for the base frame of the robot arm are entered.

The second stage of this software is used to start the robot arm operation. Before any movement of the robot arm, the first block in this stage must be ‘TRUE’. The ON/OFF commands of the servo motors in the second block of this stage are used to activate the servos of the robot arm. The servo motors must be turned ‘ON’ before the robot is moved. Otherwise, the motion data are added to the buffer and executed when the servos are turned ‘ON’.

The third stage of the software is used to determine the movements of the robot, as seen on the right of the middle row of Figure 16. For pick-and-place operations, all movements of the robot are defined to occur in Cartesian space. The pose data required to define the movement of the robot arm’s end-effector from the current position to the target pose for the desired pick movement are obtained from the image processing software running simultaneously. The fixed pose of the desired location where the robot arm is desired to release the picked object is defined here.

The code blocks in the last stage are used to stop the robot arm. To achieve this, the relevant code block must first be FALSE before the robot arm shuts down. In addition, to disable the servos in the robot arm, the servo motor blocks must be set to OFF before the robot arm shuts down. Close session code blocks are used to close the robot arm session after execution is complete or an emergency stop. This must be performed before shutting down the controller; otherwise memory leaks may occur.

### 6.2. Object Picking Pose Scenarios

**Figure 17:**
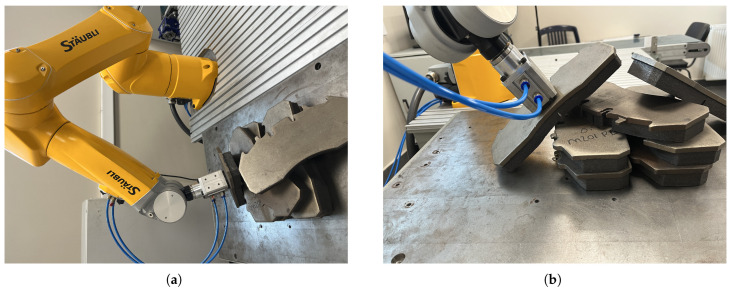
Negative slope object picking pose scenario: (**a**) Full view of negative slope configuration. (**b**) End-effector view of negative slope configuration.

**Figure 18:**
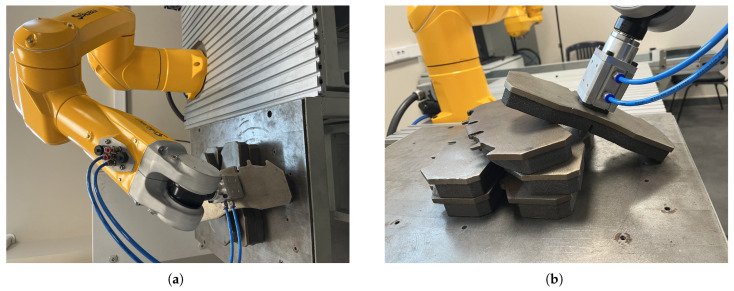
Positive slope object picking pose scenario: (**a**) Full view of positive slope configuration. (**b**) End-effector view of positive slope configuration.

**Figure 19:**
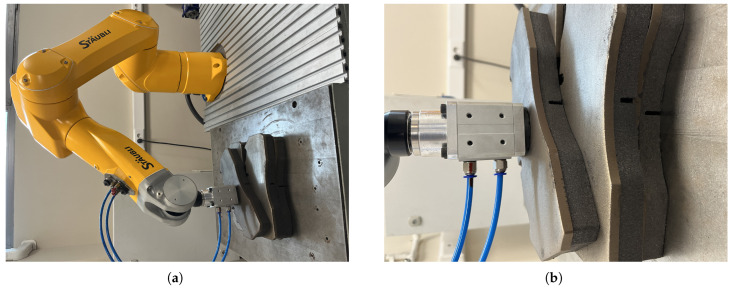
Flat horizontal-placed object picking pose scenario: (**a**) Full view of flat horizontal placing configuration. (**b**) End-effector view of flat horizontal placing configuration.

**Figure 20:**
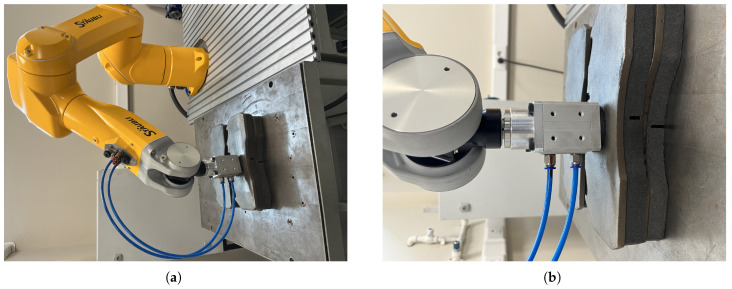
Yawed horizontal-placed object picking pose scenario: (**a**) Full view of yawed horizontal placing configuration. (**b**) End-effector view of yawed horizontal placing configuration.

In order to compare the 6D pose estimation performance of the image processing algorithms in Section 4 and Section 5, the metal plates that the robot arm will pick are placed in different poses. As seen in Figure 17, Figure 18, Figure 19 and Figure 20, the object picking pose scenarios are selected as negative slope, positive slope, flat horizontal, and yawed horizontal, respectively.

### 6.3. Results and Discussion

To perform a comparative analysis, each pick pose of the metal plates to be picked by the gripper of the robot arm for the four different scenarios mentioned above is determined according to the robot tool frame {T} (calibrated when the robot arm is in the home position according to the camera frame {C}) under the same real-world conditions. For this process, first, the gripper of the robot arm is manually brought to the center point where it should pick up the metal plate for each scenario. Then, using the forward kinematic equations given in Section 2 and the encoder sensor data at the joints of the robot arm, the desired pick poses of the metal plate relative to the robot base frame {B} are calculated. The desired reference poses of the metal plate in the object frame {O} obtained for each scenario relative to the robot tool frame {T} when the robot arm is in the home position are presented in Table 2.

Table 3 shows the poses of the metal plates calculated with respect to the robot tool frame {T} for each scenario using our analytical solution-based 6D pose estimation algorithm, which we developed based on NI LabVIEW and NI Vision.

To observe the performance of our analytical solution-based 6D pose estimation algorithm and compare it with other methods, the absolute differences between the estimated object pose values and the real object pose values are shown in Table 4.

Using the hybrid algorithms YOLO-v8 and PNP/RANSAC (four of which are EPnP, IPPE, Iterative, and AP3P and four are their hybrid variations with RANSAC), the poses of the metal plates for each scenario were calculated with respect to the robot tool frame {T}, and the estimated values with the lowest error rates are shown in Table 5.

To observe the performance of the hybrid-based method and to compare the results with the performance of our own developed analytical solution algorithm, the absolute differences between the estimated object pose values of the hybrid-based method and the real object pose values are shown in Table 6.

According to the results in Table 4, when we analyze the performance of our LabVIEW-based analytical solution algorithm, the estimation errors at the positions pxOT, pyOT, and pzOT are less than 2mm, and the estimation errors at the rotations rxOT, ryOT, and rzOT are less than 1°. With this high-precision 6D pose estimation performance, the gripper of the industrial robot arm picked the metal plates with different positions and orientations in four scenarios, and then could precisely and 100% successfully place the metal plates in the white empty slot. On the other hand, when we examine the performances of the YOLO-based PnP/RANSAC hybrid pose estimation algorithms according to the results in Table 6, the estimation errors of the positions pxOT, pyOT, and pzOT are around 2–7mm and the estimation errors of the rotations rxOT, ryOT, and rzOT are around 1.2°–3.4°. Although there are some poses estimated with relatively low error rates in estimating the 6D pose in each scenario, in general, the robot arm must pick the metal plate in exactly the right position and orientation in all six axes in order to successfully place it in the empty slot. With this low-precision 6D pose estimation performance, the gripper of the industrial robot arm could not grasp the metal plates with the correct position and orientation in four different scenarios and therefore created situations where it could not place the metal plates exactly in the white empty slot.

As reflected in the results of this study, there are several limitations that prevent YOLO-based deep learning methods and hybrid methods for 6D object pose estimation from being practically used in real-world industrial robotic pick-and-place applications. For example, these approaches require much larger trained datasets for metallic, textureless, or reflective objects that are frequently encountered in industrial environments, but creating them is labor-intensive and costly. Moreover, high-performance GPUs are required to use these methods to achieve real-time performance in industrial applications that require speed, precision, and accuracy, such as the one in this study. In contrast, our proposed analytics-based method achieves high-precision 6D pose estimation without the need for large training datasets and high-performance GPUs, significantly reducing the overhead associated with data preparation. The YOLO model required 2000 labeled images for training in our setup; this process is not necessary for the deployment of the proposed analytical method. The comparative results demonstrate that our method achieves better accuracy without the burden of data annotation or model retraining.

For the analytical method, each scenario was repeated at least 10 times, and the results were found to be highly stable, with minimal variation between trials. Therefore, the values reported in Table 3 represent typical outcomes that closely reflect average performance. For the YOLO-based hybrid methods, each of the eight pose estimation variants was also evaluated with multiple trials. Due to occasional variations in inferred key points or pose estimation algorithms, the most representative and accurate result from these repeated runs was selected for reporting in Table 5 to illustrate the practical potential of the hybrid method.

To validate the real-time applicability of the proposed method, we measured the time required for each step in the perception and control pipeline. The image acquisition time using the RGB-D camera is approximately 5 ms. Image processing, including filtering, equalization, and pose estimation, takes around 2 ms. The time to transmit the pose data to the robot controller is approximately 3 ms. Thus, the full cycle time for capturing, processing, and sending motion commands is approximately 10 ms. The robot’s motion time from pick to place (covering 1.5 m at a speed of 10 m/s) is 150 ms, which means that vision processing overhead contributes only 4.4% to the total cycle time. For comparison, the hybrid method takes approximately 50 ms for the YOLO-v8-based detection and another 200 ms for the PnP/RANSAC-based pose estimation, totaling 250 ms before the robot actuation. This makes up 62.5% of the entire pick-and-place operation, indicating a significantly higher processing delay. The comparison of timing metrics can be seen in Table 7.

In this study, orientation error comparisons were conducted using Euler angles ($r_{x}$, $r_{y}$, and $r_{z}$), which can be affected by singularities, especially when the roll and pitch angles ($r_{x}$ and $r_{y}$) approach ±90°. However, in our experimental setup, such singular configurations are not physically possible due to the geometry of the object. The metal brake pad plates used are approximately 8 mm thick and weigh 1.5 kg, making it mechanically implausible for them to assume vertical (±90°) poses on the production surface. All observed object inclinations remained within moderate angular ranges, thus avoiding gimbal lock issues.

## 7. Conclusions

This work addressed the critical challenge of high-precision 6D pose estimation for robotic pick-and-place applications in a real industrial production line. An integrated system combining a Staubli TX2-60L industrial robot, an Intel RealSense RGB-D camera, and a novel analytics-based software solution built in LabVIEW and NI Vision was successfully developed. The proposed analytical method proved to be highly effective in accurately calculating the 6D pose of metal plates in real time. In extensive experimental trials in four challenging scenarios, the system demonstrated exceptional performance, achieving position estimation errors of less than 2 mm and rotation estimation errors of less than 1°. This level of precision allowed the robot to successfully perform the pick-and-place task in all trials and accurately place the metal plates in a tight 2 mm tolerance slot.

In a comparative analysis, the developed analytical solution significantly outperformed popular hybrid methods that combined the YOLO-v8 deep learning-based object detector with various geometric-based algorithms, such as PnP and RANSAC. The hybrid method produced larger prediction errors that prevented the robot from reliably picking up and placing the metal plates. These findings highlight the value of a dedicated analytics-based approach for specific industrial applications where high accuracy and robustness are crucial. The success of this research highlights that traditional geometry-focused machine vision techniques can provide a more effective alternative to learning-based models such as YOLO and its hybrid solutions for well-defined industrial problems, providing a validated, practical, and highly reliable solution for automating complex manipulation tasks.

Although our study primarily addresses high-precision pose estimation within a semi-static industrial setting, we recognize that real-world applications frequently introduce complexities such as dynamic conveyors, moving targets, and fluctuating lighting or occlusion. However, the modular nature of our proposed method allows for adaptation to such dynamic environments. This would involve integrating elements such as high-speed imaging, motion synchronization, or predictive control. Extending this methodology to these more challenging scenarios would require custom setups, including robot-specific control strategies, customized grippers, and potentially multiple camera configurations to effectively manage occlusion and speed constraints.

The proposed method was experimentally validated using rigid and planar metallic brake pad plates due to their relevance in the target industrial application. However, the underlying analytical approach can be adapted to other types of objects with appropriate modifications to perception or manipulation systems. For example, handling reflective or non-planar objects may require different camera types (e.g., polarization or structured light sensors), lighting control, or alternative end-effectors. The current system also demonstrated robustness under cluttered scenes, with randomly oriented and overlapping objects. Future work will explore extending the method to non-metallic or deformable objects, integrating multi-view perception, and applying it in other industrial domains beyond automotive assembly lines.

## Figures and Tables

**Table 1:** The Denavit–Hartenberg parameters of the Staubli TX2-60L robot arm.

Link *i*	$\alpha_{i}$	$a_{i}$	$d_{i}$	$\theta_{i}$
1	0	0	0	$\theta_{1}$
2	−π/2	0	0	$\theta_{2}$
3	0	$a_{3}$	$d_{3}$	$\theta_{3}$
4	π/2	0	$d_{4}$	$\theta_{4}$
5	−π/2	0	0	$\theta_{5}$
6	π/2	0	$d_{6}$	$\theta_{6}$

**Table 2:** Desired object poses relative to robot tool frame {T}.

Pose (m/°)	pxOT	pyOT	pzOT	rxOT	ryOT	rzOT
Negative Slope	0.226	0.206	0.265	2.1	−46.5	42.3
Positive Slope	0.509	0.182	0.263	2.0	43.0	12.0
Flat Horizontal	0.347	0.187	0.275	0.9	1.0	121.1
Yawed Horizontal	0.380	0.192	0.306	0.8	0.7	−90.0

**Table 3:** Estimation of object poses relative to the robot tool frame {T} using LabVIEW-based analytical solution algorithm.

Pose (m/°)	pxOT	pyOT	pzOT	rxOT	ryOT	rzOT
Negative Slope	0.225	0.205	0.265	2.1	−46.9	42.0
Positive Slope	0.5105	0.181	0.263	2.5	43.8	12.5
Flat Horizontal	0.346	0.1883	0.275	1.3	1.0	120.9
Yawed Horizontal	0.3814	0.1903	0.306	1.1	1.1	−89.9

**Table 4:** Absolute differences between the estimated pose by the LabVIEW-based analytical solution algorithm and the real object pose.

Pose Error (m/°)	$\Delta_{O}^{T}p_{x}$	$\Delta_{O}^{T}p_{y}$	$\Delta_{O}^{T}p_{z}$	$\Delta_{O}^{T}r_{x}$	$\Delta_{O}^{T}r_{y}$	$\Delta_{O}^{T}r_{z}$
Negative Slope	0.001	0.001	0	0	0.4	0.3
Positive Slope	0.0015	0.001	0	0.5	0.8	0.5
Flat Horizontal	0.001	0.0013	0	0.4	0	0.2
Yawed Horizontal	0.0014	0.0017	0	0.3	0.4	0.1

**Table 5:** Estimation of object poses relative to the robot tool frame {T} using YOLO+PnP/RANSAC hybrid algorithms.

Pose (m/°)	pxOT	pyOT	pzOT	rxOT	ryOT	rzOT
Negative Slope	0.224	0.207	0.267	4.2	−46.3	43.5
Positive Slope	0.506	0.175	0.266	4.5	46.4	12.6
Flat Horizontal	0.342	0.194	0.274	1.8	1.2	121.8
Yawed Horizontal	0.376	0.194	0.299	0.9	3.1	−89.8

**Table 6:** Absolute differences between the estimated pose by the hybrid algorithm of YOLO + PnP/RANSAC and the real object pose.

Pose Error (m/°)	ΔpxOT	$\Delta_{O}^{T}p_{y}$	$\Delta_{O}^{T}p_{z}$	$\Delta_{O}^{T}r_{x}$	$\Delta_{O}^{T}r_{y}$	$\Delta_{O}^{T}r_{z}$
Negative Slope	0.002	0.001	0.002	2.1	0.2	1.2
Positive Slope	0.003	0.007	0.003	2.5	3.4	0.6
Flat Horizontal	0.005	0.007	0.001	0.9	0.2	0.7
Yawed Horizontal	0.004	0.002	0.007	0.1	2.4	0.2

**Table 7:** Comparison of timing metrics between the proposed analytical and YOLO-based hybrid methods.

Time-Consuming Steps	The Proposed Analytical Method	YOLO-Based Hybrid Method
Image Capture	5 ms	5 ms
Image Processing	2 ms	250 ms (50 ms + 200 ms)
Command Transfer	3 ms	5 ms
Total Before Robot Movement	10 ms	255 ms
Robot Motion Time	150 ms	150 ms
Total Pick-and-Place Time	160 ms	405 ms
Vision System Share	4.4%	62.5%

## Data Availability

Data are contained within the article.
